# Systematic evaluations of forensic effectiveness and genetic structures of two ethnic groups in Northwest China using a self-developed Multi-InDel panel

**DOI:** 10.1186/s41065-025-00416-5

**Published:** 2025-05-16

**Authors:** Qinglin Liang, Qiong Lan, Qinglin Liu, Xiaolian Wu, Lisiteng Luo, Chunmei Shen, Bofeng Zhu

**Affiliations:** 1 Guangzhou Key Laboratory of Forensic Multi-Omics for Precision Identification, School of Forensic Medicine, Southern Medical University, Guangzhou, Guangdong China; 2 Department of Clinical Laboratory, Nanfang Hospital, Southern Medical University, Guangzhou, Guangdong 510515 China

**Keywords:** Forensic genetics, Multi-InDel, Capillary electrophoresis, Individual identification, Kinship analysis, Population genetics

## Abstract

**Background:**

The use of compound markers has gained significant interest among forensic practitioners, due to their ability to enhance genetic marker polymorphisms by introducing new alleles. Two or more closely linked insertion/deletion (InDel) markers form a compound marker termed Multi-InDel, which offers the advantages of microhaplotype (MH) and can be genotyped using capillary electrophoresis (CE) platform. A multiplex amplification panel, including 41 Multi-InDel markers and the sex-determination locus Amelogenin, was developed and validated as an effective tool for forensic and population genetics applications.

**Methods:**

A total of 245 Kazakh and Kyrgyz samples from China were genotyped based on the 41 Multi-InDel markers to evaluate the forensic efficacy of the panel. In addition, Multi-InDel genotyping data from 28 reference populations were collected, and population genetic analyses were performed to elucidate the genetic backgrounds of Chinese Kazakh and Kyrgyz groups.

**Conclusions:**

The Multi-InDel markers demonstrated high genetic polymorphisms in Chinese Kazakh and Kyrgyz ethnic groups, indicating their suitability for forensic applications. For the two ethnic groups, the cumulative power of discrimination (CPD) values were 0.999999999999999999999999835993 and 0.999999999999999999999999717184, respectively, while the cumulative power of exclusion (CPE) values were 0.999998887418153 and 0.999999348634116, respectively. Using this Multi-InDel panel, an average of 98.82% of full sibling (FS) pairs could be distinguished from unrelated individual pairs (likelihood ratio > 1). Regarding population genetics, Chinese Kazakh and Kyrgyz groups were found to exhibit an East Asia-Europe admixed ancestry pattern, while maintaining closer genetic affinities with East Asian populations.

**Supplementary Information:**

The online version contains supplementary material available at 10.1186/s41065-025-00416-5.

## Background

The analysis of human polymorphic genetic marker is a cornerstone of forensic genetics. Currently, short tandem repeat (STR) is the predominant genetic marker used in forensic DNA identification [1, 2]. STR genotyping based on polymerase chain reaction (PCR) and capillary electrophoresis (CE) will likely remain the gold standard for forensic casework for the foreseeable future [3, 4]. However, the application of STRs has been found to have drawbacks such as a limited number of available loci, relatively high mutation rates, and long PCR amplification fragments. Single nucleotide polymorphisms (SNPs) are characterized by their high genomic density, low mutation rates, and relatively short amplicons compared to STRs, and short amplicons facilitate the genotyping of degraded biological samples [5]. In 2013, professor Kidd introduced the concept of microhaplotypes (MHs) based on SNPs at the 24th World Congress of the International Society for Forensic Genetics. MHs are defined as a single sequencing fragment with at least three haplotypes (alleles) detected [6]. When evaluated as microhaplotype, a short sequence region containing multiple SNPs within an amplicon can exhibit high level of heterozygosity. However, incorporating MHs into CE platform commonly used in forensic laboratory is challenging [7].

Insertion/deletion (InDel) genetic markers, biallelic length polymorphisms resulting from the insertion or deletion of DNA fragments, are abundant in the human genome [8, 9]. Combining the characteristics of STRs and SNPs, InDels can be genotyped on the CE platform, facilitating their implementation in routine forensic laboratories. Their lower mutation rates ensure stable inheritance, which is crucial for biogeographic ancestry inference and paternity testing [10]. Furthermore, the flexible sizes of amplification products also allow for the genotyping of degraded samples [11, 12], making InDels versatile tools in forensic genetics. Nevertheless, the information carried by the InDel as a biallelic genetic marker is limited, and the number of InDels that need to be jointly applied to achieve sufficient system efficacy is large, which also complicates the construction of multiplex amplification system. Given these considerations, researchers have endeavored to investigate the closely linked Multi-InDel genetic markers [13–17], aiming to obtain more genetic information from the same number of markers. Multi-InDel markers represent a broad type of microhaplotype and exhibit the advantages of MHs. Moreover, Multi-InDel can be genotyped on the CE platform, making them compatible with standard forensic laboratory workflow. Considering the above advantages, a multiplex amplification panel was constructed, comprising 41 Multi-InDel markers and sex-determination locus Amelogenin. This panel includes 82 InDel markers, with each Multi-InDel consisting of two closely linked InDels. In addition, the panel has been validated as an effective tool in previous studies [18–20].

According to Chinese seventh national population census (https://www.stats.gov.cn/sj/pcsj/rkpc/7rp/zk/indexch.htm), Chinese Kazakh and Kyrgyz ethnic groups number over 1.56 million and 200,000, respectively, and are recognized as significant ethnic minorities in China. These two groups primarily inhabit northwestern China, which is located at the crossroad of the Eurasian continent and is historically connected to the Silk Road. The Silk Road is a major corridor linking East Asia, Central Asia and Europe, and plays an important role in economic exchange and population migration. As long-term settled groups in the region, Chinese Kazakh and Kyrgyz groups are key to understanding the history of genetic exchange between East and West Eurasia. Recent advances have revealed that Chinese Kazakh and Kyrgyz groups exhibit considerable East-West admixture, providing deeper insights into the complex genetic relationships between Western and East Asian populations [21, 22]. However, forensic research on these two ethnic groups remains limited, particularly in the application of Multi-InDel genetic markers. Therefore, this study utilizes an self-developed panel containing 41 Multi-InDel markers to systematically evaluate its forensic applicability in Chinese Kazakh and Kyrgyz ethnic groups, as well as to explore their genetic structures and backgrounds through population genetic analyses.

## Methods and materials

### Sample collection

A total of 245 blood samples were collected, including 145 Kazakh and 100 Kyrgyz individuals. The participants self-reported good health, were not related within three generations, and had no history of intermarriage or migration. Prior to their participation, all volunteers were informed of the purpose of this research and provided with a written informed consent form to sign. Our sample collection and genotyping protocol have been reviewed and approved by the Ethics Committees of Southern Medical University and Xi’an Jiaotong University (No. 2019–1039). For population genetics analysis, the Multi-InDel genotyping data of 26 populations from five continents (Africa, America, Europe, East Asia, and South Asia) were acquired from the 1000 Genomes Project Phase 3 [23], as well as previously published 41 Multi-InDel markers genotyping data from Chinese Manchu and Mongolian groups [19]. All relevant information pertaining to the populations was provided in the Table S1.

### DNA extraction and quantification

All blood samples were stored on FTA cards and dried before extraction. A diameter of 1 mm bloodstain sample was prepared for DNA extraction for each sample FTA card. Genomic DNA was extracted from the bloodstain sample according to the instruction following the Chelex-100 method [24]. DNA 9948 and deionized sterile water were used as positive and negative controls, respectively. The concentration and purity of template DNA were determined using a NanoDrop 1000 spectrophotometer (Thermo Fisher Scientific Waltham, MA, USA).

### PCR amplification, capillary electrophoresis, and genotyping

The 41 Multi-InDel markers and an Amelogenin marker were designed into four lanes, labeled with four different dyes. Table S2 lists the location and fluorescence details of the panel. DNA amplification was performed using a GeneAmp^®^ PCR System 9700 thermal cycler (Applied Biosystems, Foster City, California, USA). The amplification system had a total volume of 10 µL, consisting of 2 µL 2.0× master mix, 1 µL (1 ng) template DNA, 2 µL 1.0× primer mix, and 5 µL nuclease-free water. The diluted amplified product was detected via CE on a 3500xL Genetic Analyzer (Applied Biosystems, Foster City, California, USA). The DNA profiles of the 41 Multi-InDel markers were analyzed by GeneMapper^®^ ID-X 1.3 software. The genotype of each Multi-InDel marker was determined based on the genotype of two InDel loci. Allele 0 represents simultaneous deletion fragments at both InDel loci, while an allele 3 represents the simultaneous insertion fragments at both InDel loci. Allele 1 or 2 represents one InDel locus as the insertion allele and the other locus as the deletion allele. In this study, allele 1 represents a relatively short amplicon, while allele 2 represents a relatively long amplicon. Since the two InDel loci in each of the selected Multi-InDel markers have disparate insertion or deletion fragment lengths, the lengths of the allele 1 and allele 2 amplicons differed.

### Data analysis

GenALEx (version 6.5) [25] and GENEPOP (version 4.0.10) [26] were employed to analyze Hardy-Weinberg equilibrium (HWE) and linkage disequilibrium (LD) of Multi-InDel markers in Kazakh and Kyrgyz groups. And *p*-values from HWE and LD tests were adjusted using Bonferroni’s correction. Gene frequencies and forensic parameters, including polymorphism information content (PIC), probability of match (PM), power of discrimination (PD), observed heterozygosity (Hobs), and power of exclusion (PE), were calculated for 41 Multi-InDel markers using the STRAF (version 2.1.5) online tool [27]. The relevant forensic parameters were visualized in a split violin plot for the two groups using the ggunchained package of the *R* software (version 4.2.1). The informativeness for assignment (*I*_n_) was calculated using the Infocalc (version 1.1) [28], which is used to quantify the information content of Multi-InDel markers in distinguishing ancestral origions. Gene frequencies and *I*_n_ values were visualized using the online website ChiPlot (https://www.chiplot.online/).

In order to assess the efficacy of the Multi-InDel panel for kinship analysis, 10,000 full sibling (FS) pairs, 10,000 half sibling (HS) pairs, and 10,000 pairs of unrelated individuals were simulated by the Familias 3 software [29], based on the gene frequencies for the 41 Multi-InDel markers. Familias 3 was also used to calculate likelihood ratios (LR) for different relationships. The prosecution hypothesis (H0) posited that two individuals are either HS or FS, whereas the defense hypothesis (H1) posited that they are unrelated individuals. The LR distributions for these relationships were visualized using *R* software (version 4.2.1).

The paired *Nei*’s genetic distances (*D*_A_ distances) [30] and fixation index (*F*_ST_) values for a total of 30 populations were obtained using the Dispan program and GenAIEx (version 6.5), respectively. Subsequently, *D*_A_ distances and *F*_ST_ values were visualized using the ggplot2 package of *R* software (version 4.2.1). On the basis of the pairwise *D*_A_ distances, a neighbor-joining (NJ) phylogenetic tree was constructed using MEGA 11 software [31] based on the neighbor-joining method and plotted using ChiPlot. Furthermore, principal component analysis (PCA), t-distributed stochastic neighbor embedding (t-SNE) and uniform manifold approximation and projection (UMAP) were used to visualize the genetic relationships of the two studied groups and 28 reference populations, which were conducted at both the population and individual levels using the Rtsne and umap packages in *R* software (version 4.2.1).

Additionally, the STRUCTURE (version 2.3.4) [32] was utilized to assess the Bayesian clustering of genotype data in the two studied groups and reference populations. The STRUCTURE program was executed with the following parameters: *K* = 2–7 (15 replicates per *K*), and 10,000 MCMC iterations. Subsequently, the genetic components for each *K* value were plotted in a stacked format using the Distruct (version 1.1) [33] software. STRUCTURE HARVESTER [34] was used to evaluate and visualize the likelihood values and to estimate *ΔK*. The merged Q matrices for the 15 replications of optimal *K* values were obtained by the CLUMPP (version 1.1.2) [35], and plotted based on CLUMPP results using the AncestryPainterV2 package in *R* (version 4.2.1).

## Results

### HWE and LD analyses of 41 Multi-InDel markers in the Kazakh and Kyrgyz ethnic groups

In the HWE analysis, the MI38 marker of both Kazakh group (*p* = 0.000) and Kyrgyz group (*p* = 0.001) deviated from HWE after Bonferroni's correction (*p* = 0.05/41), which was consistent with the previous research [18, 20]. Therefore, the MI38 marker was excluded from the subsequent analysis. HWE and LD analyses were performed on the remaining 40 Multi-InDel markers. After Bonferroni's correction, 40 markers in both groups were in accordance with HWE (*p* > 0.05/40), and no significant association was found between paired markers, indicating a state of linkage equilibrium (*p* > 0.05/780) in 40 markers, in these two groups.

### Gene frequency distributions and forensic parameters of Multi-InDel markers in two groups

**Fig. 1:**
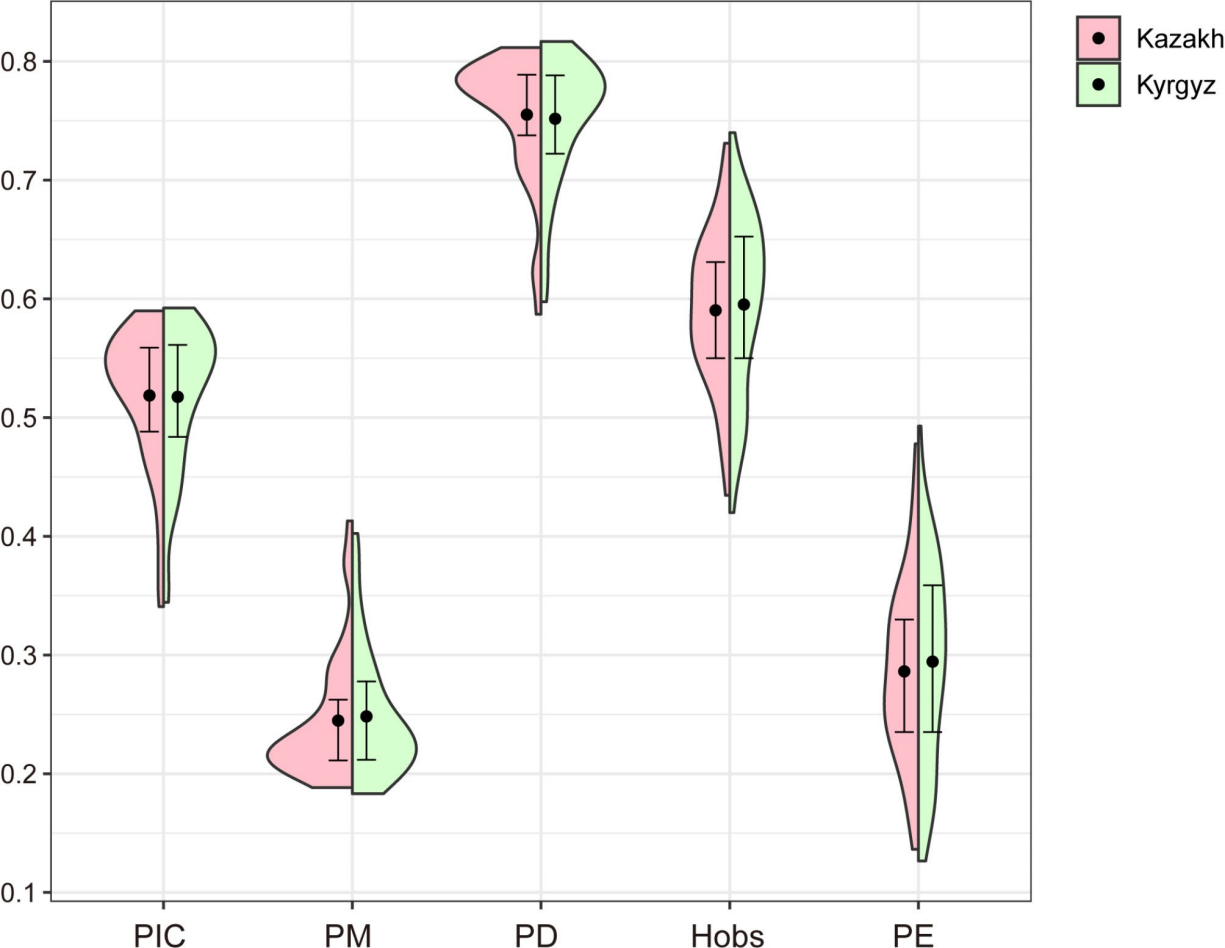
Forensic parameters of the 40 Multi-InDel markers in Chinese Kazakh and Kyrgyz groups. In this split violin plot, the dots represent the mean values of forensic parameters, and the horizontal lines at both ends of the vertical lines represent the upper and lower quartiles of the data. PIC, polymorphism information content; PM, probability of match; PD, power of discrimination; Hobs, observed heterozygosity; PE, power of exclusion

We calculated the gene frequencies and forensic parameters of 40 Multi-InDel markers in the two studied groups, respectively. The gene frequency distributions are shown in the Fig. S1 and Table S7, and most of the 40 Multi-InDel markers in two groups have three alleles. As shown in Fig. 1 and Table S3, in the Kazakh group, the forensic parameters range as follows: PIC from 0.3408 (2MI16) to 0.5898 (2MI58), PM from 0.1883 (MI32) to 0.4130 (2MI16), PD from 0.5870 (2MI16) to 0.8117 (MI32), Hobs from 0.4345 (2MI16) to 0.7310 (2MI58), and PE from 0.1363 (2MI16) to 0.4779 (2MI58). In the Kyrgyz group, these parameters show the following ranges: PIC from 0.3444 (2MI16) to 0.5923 (2MI58), PM from 0.1832 (2MI17) to 0.4024 (2MI16), PD from 0.5976 (2MI16) to 0.8168 (2MI17), Hobs from 0.4200 (2MI16) to 0.7400 (2MI07), and PE from 0.1266 (2MI16) to 0.4928 (2MI07). In addition, the cumulative matching probability (CPM), cumulative power of discrimination (CPD), and cumulative power of exclusion (CPE) of 40 markers in the Kazakh and Kyrgyz groups are 1.64007E-25 and 2.82816E-25; 0.999999999999999999999999835993 and 0.999999999999999999999999717184; 0.999998887418153 and 0.999999348634116, respectively, indicating that the panel could be a powerful tool for individual identification and paternity testing in the two groups.

### The effectiveness of Multi-InDel panel in identifying full siblings and half siblings of two groups

**Fig. 2:**
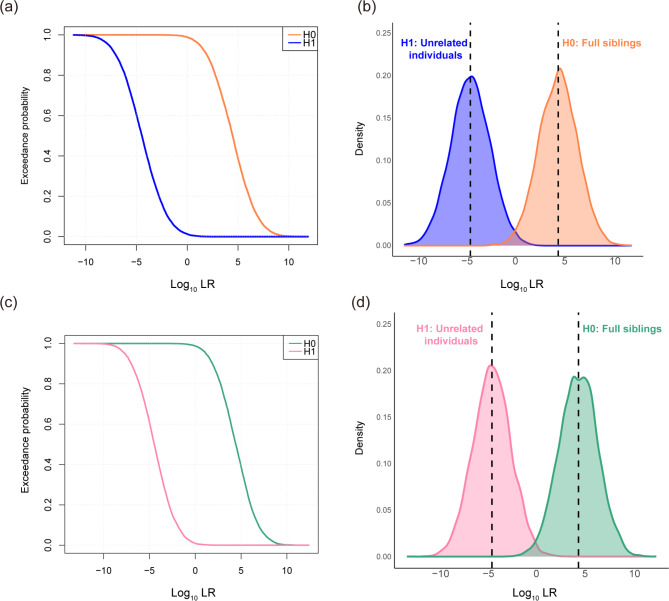
Results of simulating full sibling (FS) pairs and unrelated individual pairs based on gene frequencies of 40 Multi-InDel markers in Chinese Kazakh and Kyrgyz ethnic groups. (**a**) Exceedance probability curve for Log_10_LR of FS and unrelated individuals in Chinese Kazakh group; (**b**) Kernel density profile of Log_10_LR for 10,000 FS pairs and 10,000 unrelated individual pairs simulated in Kazakh group; (**c**) Exceedance probability curve for Log_10_LR of FS and unrelated individuals in Kyrgyz group; (**d**) Kernel density profile of Log_10_LR for 10,000 FS pairs and 10,000 unrelated individual pairs simulated in Kyrgyz group

We evaluated the forensic efficacy of the Multi-InDel panel for identifying FS and HS pairs in the Kazakh and Kyrgyz groups. The Log_10_LR distributions between FS pairs and unrelated pairs in the two groups are shown in Fig. 2. The accuracy and false positive rates for identifying FS and HS using LR thresholds are detailed in Table S4. Using the 40 Multi-InDel markers, a similar ability to distinguish FS pairs in the Kazakh and Kyrgyz groups was observed. When LR = 1, 98.89% and 98.74% of FS pairs could be differentiated from unrelated individual pairs in the two groups, with false positive rates of 1.27% and 0.95%, respectively. When the LR thresholds were set at 10, 100, 1,000, and 10,000, the average accuracies of the two groups were 96.03%, 89.12%, 76.34%, and 58.59%, respectively, while the corresponding average false positive rates were 0.27%, 0.04%, 0.00%, and 0.00%, respectively. However, the Multi-InDel panel was less effective in distinguishing HS pairs from unrelated individual pairs. When the LR thresholds were set at 1, 10, and 100, the average accuracies for identifying HS pairs in the two groups were 86.65%, 51.19%, and 1.68%, respectively.

### Genetic distances among the two studied groups and 28 reference populations

**Fig. 3:**
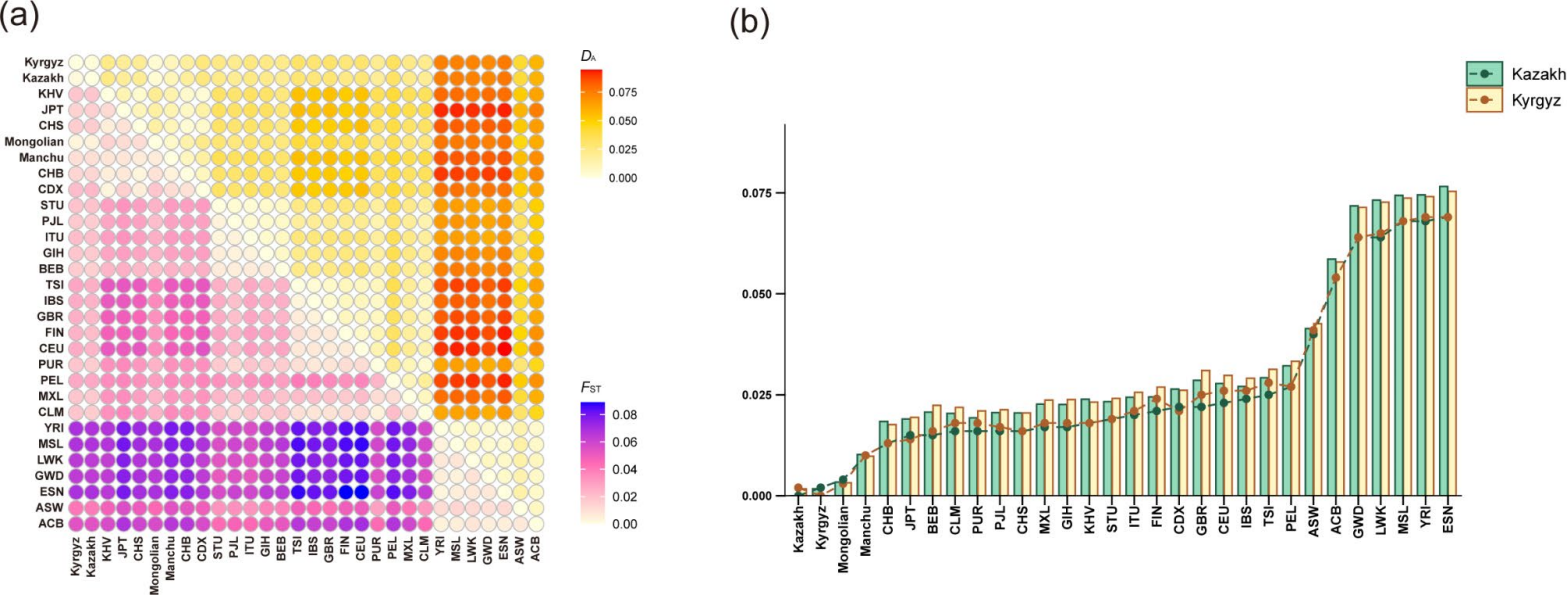
*F*_ST_ values and *D*_A_ values for paired groups. (**a**) Heatmap of *F*_ST_ values (lower left corner) and *D*_A_ values (upper right corner) among paired groups. (**b**) *F*_ST_ values (line chart) and *D*_A_ values (histogram) among the two studied groups and other reference populations. KHV, Kinh in Ho Chi Minh City, Vietnam; JPT, Japanese in Tokyo, Japan; CHS, Southern Han Chinese, China; Mongolian; Manchu; CHB, Han Chinese in Beijing, China; CDX, Chinese Dai in Xishuangbanna, China; STU, Sri Lankan Tamil from the UK; PJL, Punjabi from Lahore, Pakistan; ITU, Indian Telugu from the UK; GIH, Gujarati Indian from Houston, Texas; BEB, Bengali from Bangladesh; TSI, Toscani in Italia; IBS, Iberian Population in Spain; GBR, British in England and Scotland; FIN, Finnish in Finland; CEU, Utah Residents (CEPH) with Northern and Western European Ancestry; PUR, Puerto Ricans from Puerto Rico; PEL, Peruvian in Lima, Peru; MXL, Mexican Ancestry from Los Angeles, USA; CLM, Colombians from Medellin, Colombia; YRI, Yoruba in Ibadan, Nigeria; MSL, Mende in Sierra Leone; LWK, Luhya in Webuye, Kenya; GWD, Gambian in Western Divisions in the Gambia; ESN, Esan in Nigeria; ASW, African Americans from the Southwest USA; ACB, African Caribbean in Barbados

The *F*_ST_ value and *D*_A_ distance can be used to measure the degree of genetic differentiation between paired populations. Smaller value indicates a lower degree of genetic differentiation between the two populations. The *F*_ST_ values and *D*_A_ distances among the two groups and 28 reference populations are visualized in Fig. 3 and Table S5-S6. The African populations are the most genetically distant from the other four continents. Among these populations, the two studied groups display closer genetic relationships with East Asian populations. The average *F*_ST_ values between the two studied groups and seven East Asian populations are only 0.0125 (Kazakh) and 0.0121 (Kyrgyz). These values are significantly lower than the average *F*_ST_ values between the two studied groups and African populations, which are 0.0610 (Kazakh) and 0.0614 (Kyrgyz). It is noteworthy that the Kazakh and Kyrgyz groups exhibit the closest genetic relationship, showing the smallest *F*_ST_ value of 0.0020. *D*_A_ values corroborate these findings, with the average *D*_A_ values between the two groups and East Asian populations being 0.0154 (Kazakh) and 0.0152 (Kyrgyz), which are also far lower than the average *D*_A_ values between the two groups and other continental populations. The two groups still exhibit the smallest genetic distance from each other, with a *D*_A_ of 0.0017. These results highlight the close genetic relationship between the Kazakh and Kyrgyz groups, as well as their closer genetic relationships to East Asian populations compared to other continental populations.

### NJ phylogenetic tree construction of the two studied groups and 28 reference populations based on pairwise *D*_A_ values

**Fig. 4:**
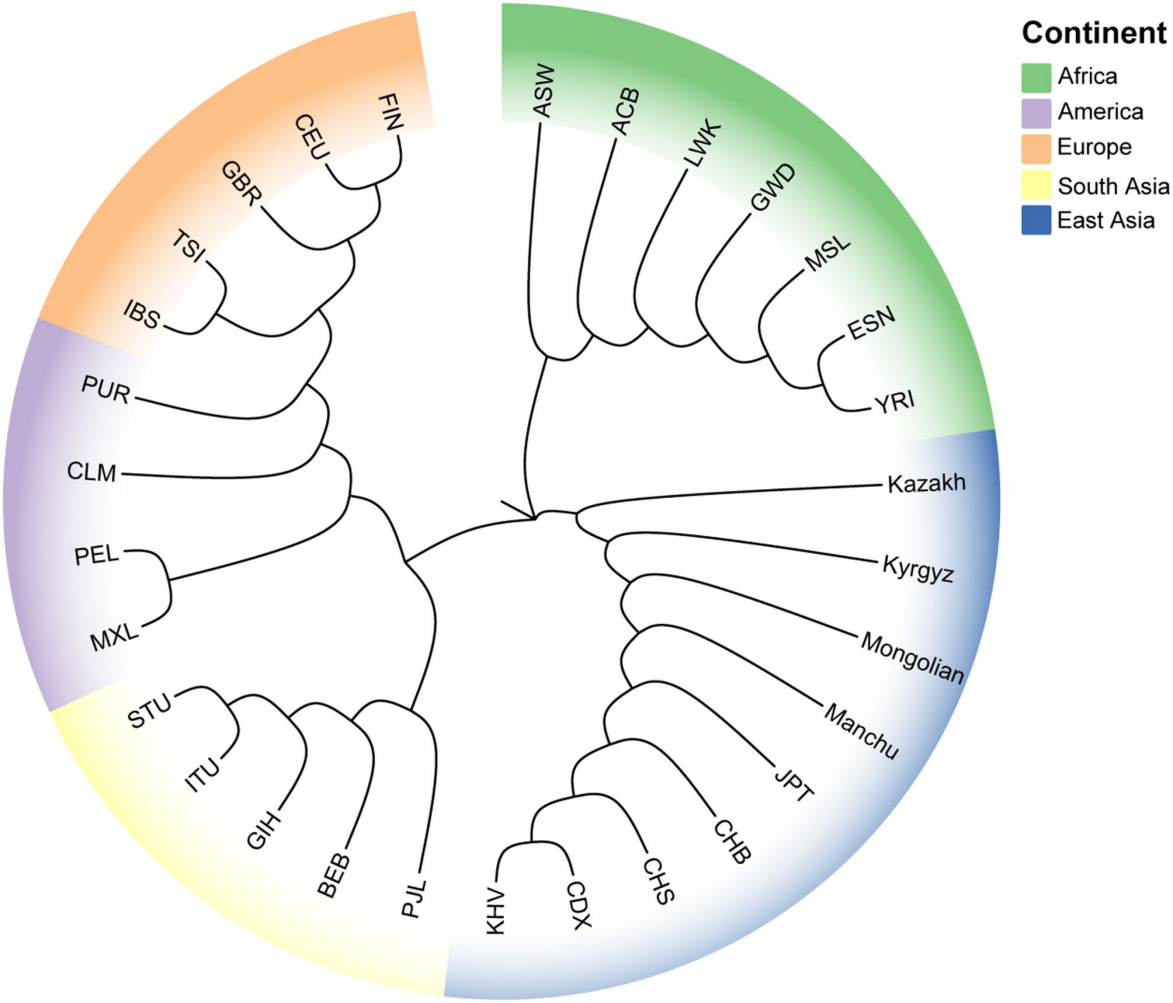
NJ phylogenetic tree of the two studied groups and 28 reference populations based on paired *D*_A_ values

The Fig. 4 presents a NJ phylogenetic tree based on paired *D*_A_ values. The NJ phylogenetic tree shows that the 30 populations cluster into three distinct evolutionary branches. African populations form a separate evolutionary branch. East Asian populations form another distinct evolutionary branch. Within this branch, two clades are identified. One clade includes the Kazakh group and the other clade consists of the Kyrgyz, CHB, CHS, CDX, Manchu, Mongolian, JPT, and KHV populations. Whereas, European, American, and South Asian populations cluster into a single branch. And this branch is further subdivided into two clades. And one clade consists of European and American populations; the other clade consists of South Asian populations. While, the four American populations did not form a separate branch due to the presence of genetically mixed ancestral components.

### *I*_n_ values for two studied groups based on 40 Multi-InDel genotype data

**Fig. 5:**
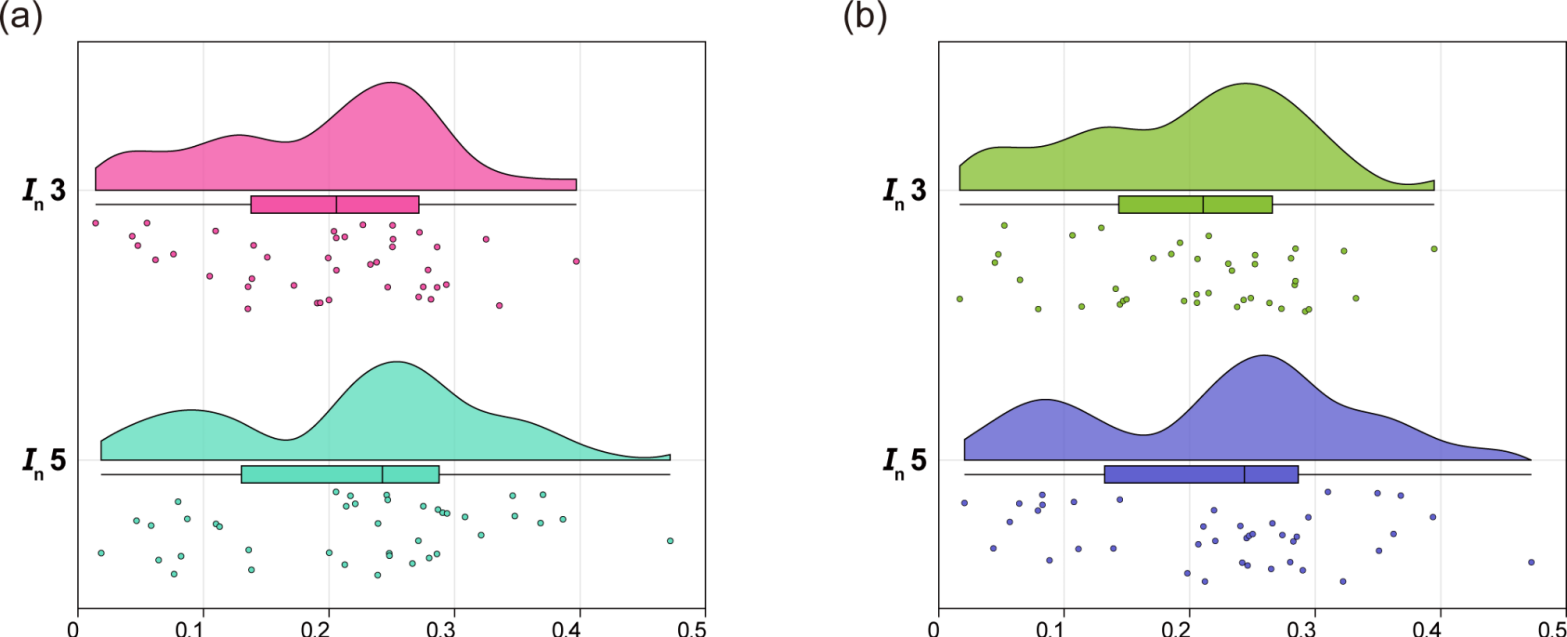
*I*n values based on 40 Multi-InDel markers when distinguishing between two studied groups and the populations from three or five continents. (**a**) *I*n values of the 40 Multi-InDel markers for distinguishing the Kazakh group from three intercontinental populations (*I*n3) and five continental populations (*I*n5); (**b**) *I*n values of the 40 Multi-InDel markers for distinguishing the Kyrgyz group from three intercontinental populations (*I*n3) and five intercontinental populations (*I*n5)

The capacity of the 40 Multi-InDel markers to provide information regarding an individual’s ancestral information in the two studied groups and the reference populations was evaluated using parameter *I*_n_. A higher *I*_n_ value indicates a stronger ability of Multi-InDel marker to infer the ancestry of unknown individual. We calculated the *I*_n_ values for the 40 Multi-InDel markers to distinguish the two studied groups from three (Africa, Europe, and East Asia) and five (Africa, Europe, East Asia, South Asia, and America) continents, denoted as the parameters *I*_n_3 and *I*_n_5, respectively. As demonstrated in Fig. 5 and Table S8, the *I*_n_ values of most genetic markers are concentrated between 0.2 and 0.3. Additionally, 85% (34/40) and 80% (32/40) of the Multi-InDel markers exhibited *I*_n_3 and *I*_n_5 values exceeding 0.1 in the Kazakh and Kyrgyz groups. Notably, *I*_n_5 and *I*_n_3 were observed to be greater than 0.3 for 2MI54, 2MI49, and MI26 markers in both studied groups, indicating their efficacies in providing meaningful insights into the ancestral backgrounds of the two groups.

### Dimensionality reduction analyses for 40 Multi-InDel markers of the two studied groups and 28 reference populations

**Fig. 6:**
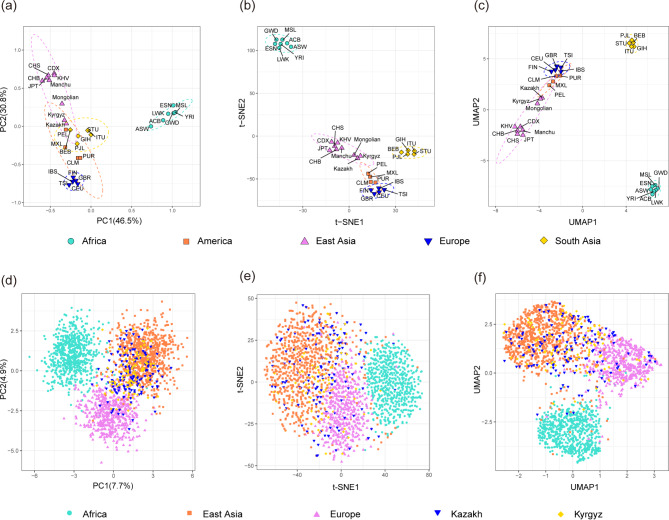
Population-level and individual-level PCA, t-SNE and UMAP dimensionality reduction analyses based on 40 Multi-InDel markers. (**a**) Population-level PCA of two studied groups and the reference populations at PC1 and PC2; (**b**) Population-level t-SNE of two studied groups and the reference populations; (**c**) Population-level UMAP of two studied groups and the reference populations; (**d**) PCA of the overall individuals from three continents (Africa, Europe, and East Asia); (**e**) t-SNE of the overall individuals from three continents (Africa, Europe, and East Asia); (**f**) UMAP of the overall individuals from three continents (Africa, Europe, and East Asia)

To facilitate comprehension and analyses of the data structures and patterns, we projected high-dimensional data into a two-dimensional space. Population-level and individual-level PCA, t-SNE, and UMAP dimensionality reduction analyses were conducted and visualized for the two studied groups and 28 reference populations based on gene frequencies and raw genotypes of 40 Multi-InDel markers, respectively. These results are visualized in Fig. 6. At the population level, the first two principal components of PCA explained 77.3% of the total variance. The Fig. 6a depicts the 30 populations, which are roughly clustered into four clusters, i.e. the African populations cluster on the right (green), East Asian populations cluster on the top left (purple), European populations cluster on the bottom left (blue), and South Asian populations cluster in the center (yellow) in the PCA. In the Fig. 6b, the result of t-SNE indicates that populations from the same continent tend to cluster with each other and there is no overlap in distribution, and further distinguishes the South Asian populations compared to PCA. In the Fig. 6c, the distribution pattern of UMAP is roughly similar to that of t-SNE, but there is overlap. The genetic relationships of the two studied groups were further analyzed in relation to the East Asian, European, and African populations by individual-level dimensionality reduction with the genotypes of 40 Multi-InDel markers. The Fig. 6d-f illustrate that a total of 2,213 individuals from three continents (East Asia, Africa and Europe) are divided into three clusters. Of these, 245 Kazakh and Kyrgyz individuals are superimposed on the East Asian and European individuals, with a greater degree of overlap observed between them and the East Asian individuals. This pattern supports closer genetic affinities between the studied groups and the East Asian populations.

### Population genetic structure analyses among the two studied groups and 28 reference populations

**Fig. 7:**
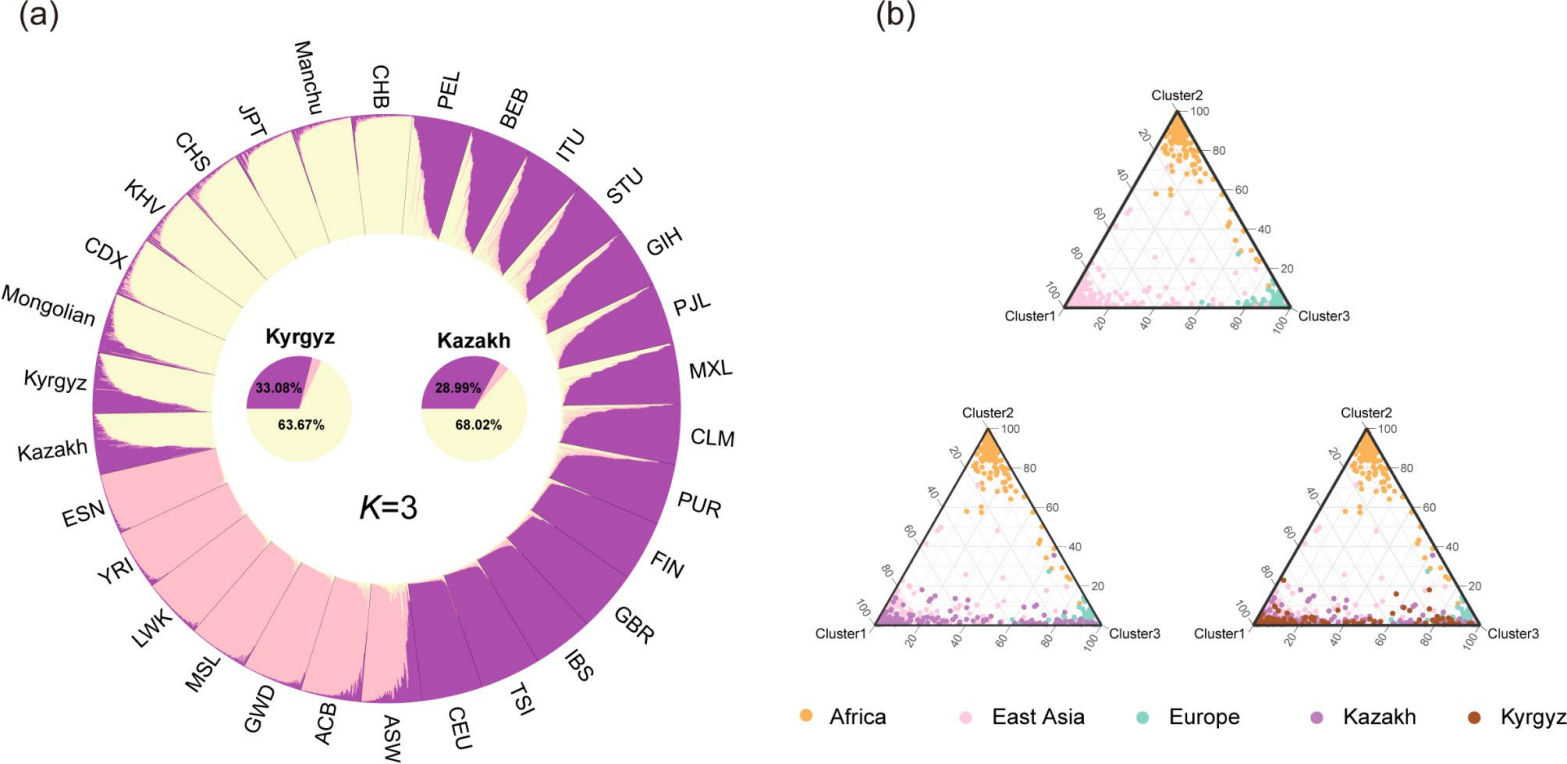
Genetic structure analysis plots of the two studied groups and 28 reference populations. (**a**) Individual-level ancestral structures of the 30 populations when *K* = 3, and the pie chart of ancestral compositions of Kazakh and Kyrgyz group. (**b**) Triangular clustering diagram of African, East Asian, and European individuals at *K* = 3, with stepwise addition of the Kazakh and Kyrgyz individuals

We conducted individual-level and population-level ancestral component analyses based on the genotyping data of 40 Multi-InDel markers to assess the population structures of the two studied groups and 28 reference populations. The results for *K* = 2–7 are depicted in Fig. S2, which presents the stacked plots at both the individual and population levels. When *K* = 2, the genetic structure analysis identifies African and non-African ancestral components. As the *K* value increases from three to five, the Europe, East Asia, South Asia and America are distinguished by exhibiting unique ancestral components. And the two studied groups (Kazakh and Kyrgyz) and East Asian populations exhibit similar genetic structures. The optimal *K* value of three was determined according to STRUCTURE HARVESTER. Fig. 7 illustrates the individual-level stacked plot and ternary diagram for the optimal *K* value (*K* = 3). Fig. 7a shows that the maximum ancestral components of the two studied groups are similar to those of the East Asian populations. The estimated East Asian ancestry components of the Kazakh and Kyrgyz groups are 63.67% and 68.02%, respectively, and a certain percentage of European ancestry components also detected, accounting for 33.08% and 28.99% of their genetic compositions, respectively.

Because the South Asian and American populations in the 1000 Genomes Project have genetic characteristics of mixed origins which are not conducive to analytical interpretation of the results, these two intercontinental populations were not included in the triangular clustering analysis. The Fig. 7b shows that the 40 Multi-InDel markers can effectively discriminate African, European and East Asian individuals. Both Kazakh and Kyrgyz individuals partially overlap with East Asian and European individuals and share similar genetic structures.

## Discussion

The Multi-InDel marker was first proposed by Huang et al. in 2014 [13]. This marker exhibits the characteristics of a low mutation rate and a short amplicon, similar to that of MH. Of particular significance is its ability to be genotyped using the CE method, which is a common practice in forensic laboratory. In this study, 145 Kazakh and 100 Kyrgyz unrelated healthy individuals from China were genotyped using a panel containing 41 Multi-InDel markers and a sex-determination locus Amelogenin. Subsequently, the forensic parameters and genetic polymorphisms of the 41 Multi-InDel were subjected to comprehensive assessment. Furthermore, genotyping data on 41 Multi-InDel genotypes from 28 reference populations were collected in order to explore the genetic differentiations and relationships between the two studied groups and other reference populations. We found that the Multi-InDel panel qualified as an effective tool for individual identification and paternity testing of Chinese Kazakh and Kyrgyz groups, as well as for full sibling kinship identification. In addition, we provided evidence for the genetic relationships of Chinese Kazakh and Kyrgyz groups with East Asian and European populations. 

Following Bonferroni's correction, 40 out of 41 Multi-InDel markers in two studied groups demonstrated HWE, with the exception of MI38. This deviation may be attributed to the fact that the marker was purely summed and the gene frequencies were not balanced in most of the samples in both studied groups. The 40 markers were in linkage equilibrium with each other, indicating their mutual independence. Consequently, the product rule can be applied to calculate the cumulative probabilities for this Multi-InDel panel, specifically CPD, CPE and CPM. 

The Multi-InDel markers have been developed with the objective of enhancing genetic polymorphisms through the introduction of new alleles. Most of the 40 Multi-InDel markers in two studied groups have three alleles (Fig. S1), exhibiting higher polymorphisms than InDel. Genetic markers with PIC values >0.5 were considered to possess high information content [36]. As listed in Table S3, the mean PIC values of 40 Multi-InDel markers are 0.5186 and 0.5175 in the Kazakh and Kyrgyz groups, respectively, and both of them have 72.5% (29/40) markers with PIC values greater than 0.5. These results also indicate high polymorphisms of these markers. The CPD and CPE for the 40 Multi-InDel markers in the Kazakh and Kyrgyz ethnic groups were 0.999999999999999999999999835993 and 0.999999999999999999999999717184; 0.999998887418153 and 0.999999348634116, respectively. This suggested that analyses of the 40 Multi-InDel markers were eligible in individual identification and paternity testing. The 40 Multi-InDel markers demonstrated higher CPD and CPE values than the 20 Multi-InDel markers reported by Huang et al. [13], the 17 Multi-InDel markers by Qu et al. [16], and the 20 Multi-InDel markers by Liu et al. [17]. This may indicate that this panel is more effective for individual identification and paternity testing, though the higher efficacy may be attributed to the fact that the Multi-InDel panel contains more markers than the other panels. Additionally, the capability of this Multi-InDel panel is essentially equivalent to the STR panel for the two groups that have been studied [37–39]. Furthermore, the 40 Multi-InDel markers were employed for the identification of FS, HS and unrelated individual pairs. When the LR threshold was set at 1, the 40 Multi-InDel markers yielded meaningful conclusions in the context of FS identification cases, while its ability to differentiate HS from unrelated individuals was relatively limited. 

In forensic practice, ancestry information can provide crucial insights that narrow the scope of an investigation when DNA database matches are unavailable or there is a lack of reliable eyewitness testimony. Multi-InDel markers have the potential to serve as ancestry inference markers [14]. Firstly, the populations from the same continent have small *F*_ST_ and *D*_A_ values, and the Kazakh and Kyrgyz groups exhibited the smallest *F*_ST_ and *D*_A_ values with East Asian populations, in particular the genetic distance between the Kazakh and Kyrgyz groups is the smallest. The NJ phylogenetic analysis also confirmed that there were more genetic correlations between the two studied groups and East Asian populations. Secondly, the applications of PCA, t-SNE and UMAP revealed the aggregation of populations from the same continent into a single cluster. Moreover, the latter two methods demonstrated superior performance in terms of population-level distribution effects. The t-SNE better addresses the crowding problem in high-dimensional data through its nonlinear dimensionality reduction and retention of local structure, which makes the distribution of the reduced data in the low-dimensional space more uniform and intuitive. And UMAP focuses on preserving the global structure, resulting in smaller distances within the cluster. At the individual level, the three continents (Africa, Europe, and East Asia) were well differentiated, and individuals from two groups were distributed between East Asian and European individuals, with a greater overlap observed between them and the East Asian individuals. This suggests that they were more genetically related to East Asian populations. Furthermore, the results of the population structure analyses indicated that, within the range of *K* values between two and five, populations from five continents were progressively differentiated, and the genetic compositions of the two groups are comparable to East Asian populations. At the optimal *K* value of three, the East Asian ancestry components of Kazakh and Kyrgyz groups were 63.67% and 68.02%, with European ancestry components of 33.08% and 28.99%, respectively. The triangular clustering diagram also showed that individuals from these two groups were distributed between the East Asian and European individuals. This suggests stronger genetic affinities between these two groups and East Asian populations, as well as the possibility of gene admixture with East Asian and European populations. It should be noted, however, that only five populations in East Asia and two previously studied groups were selected as reference populations in this study, and cannot fully represent the entire East Asian populations. The same is true for the reference populations in Africa, America, Europe and South Asia. Therefore, more populations are needed in future study to further confirm the robustness of the panel.

Research based on autosomal STRs and Y-SNPs/STRs had demonstrated that the genetic component of Kyrgyz group was similar to both East Asian and European populations [40]. Furthermore, genome-wide SNP studies of present-day Chinese Kazakh [21], Mongolian [41], and Kyrgyz [42] groups also support their East Asia-Europe mixing pattern. Additionally, studies of autosomal STRs [38, 43] and DIPs [44] suggested that the Kyrgyz and Kazakh groups exhibited close genetic affinities. These findings corroborate our results. In the modern era, the Kazakh group was compelled to migrate in significant numbers to the Ili, Tacheng and Altai regions of northwest China. They subsequently continued to migrate to the northern foothill of the Tianshan Mountain, which altered and shaped the pattern of ethnic distribution and ethnic relation in the region [45]. The Kyrgyz group first inhabited the Yenisei River basin. Due to war and other factors, they experienced five westward migrations between the Western Han Dynasty and the middle of the Qing Dynasty, reaching as far as the Western Tian Shan and Central Asia [46]. Currently, they reside in mixed communities with the Kazakh, Uyghur, Mongolian, and Han Chinese groups. Both studied groups reside in the northwestern region of China, which was the route of ancient Silk Road, an important link for the exchange of good, plant, animal, and idea between the people of East Asia and Europe [47]. Due to their special geographic location and multiethnic gathering, population movements and intermarriages were inevitable [48], which may explain the fact that the two studied groups in our results consisted mainly of genetic components from East Asian and European populations and had the smallest genetic distance between them.

## Conclusions

In this study, a total of 245 individuals from Chinese Kazakh and Kyrgyz ethnic groups were conducted forensic and population genetic analyses using a self-developed panel containing 41 Multi-InDel markers. The results showed that these 40 Multi-InDel markers (except MI38) can be used as effective tools for individual identification and paternity testing of two studied groups, and play a potential role in full sibling identification. In addition, population genetic analyses further elucidated the East Asia-Europe admixed ancestry components in the Kazakh and Kyrgyz groups, while demonstrating closer genetic affinities with East Asia populations. Ancestral components of five intercontinental populations can be preliminarily inferred based on the 40 Multi-InDel markers. At the same time, we expanded the genetic dataset of these two ethnic groups. Overall, this study demonstrates that the Multi-InDel panel can play an important role in forensic application and ancestry inference. Future investigations with more groups are needed to confirm the robustness of the panel.

## Electronic supplementary material

Below is the link to the electronic supplementary material.

Supplementary Material 1

Supplementary Material 2

Supplementary Material 3

## Data Availability

The raw genotype data used and analyzed during the current study are available from the corresponding author on reasonable request.
