# Supplementary figures and images for: Systematic evaluations of forensic effectiveness and genetic structures of two ethnic groups in Northwest China using a self-developed Multi-InDel panel
Source: Hereditas. 2025 May 16;162:80. doi: 10.1186/s41065-025-00416-5 (PMC12083099; doi:10.1186/s41065-025-00416-5)

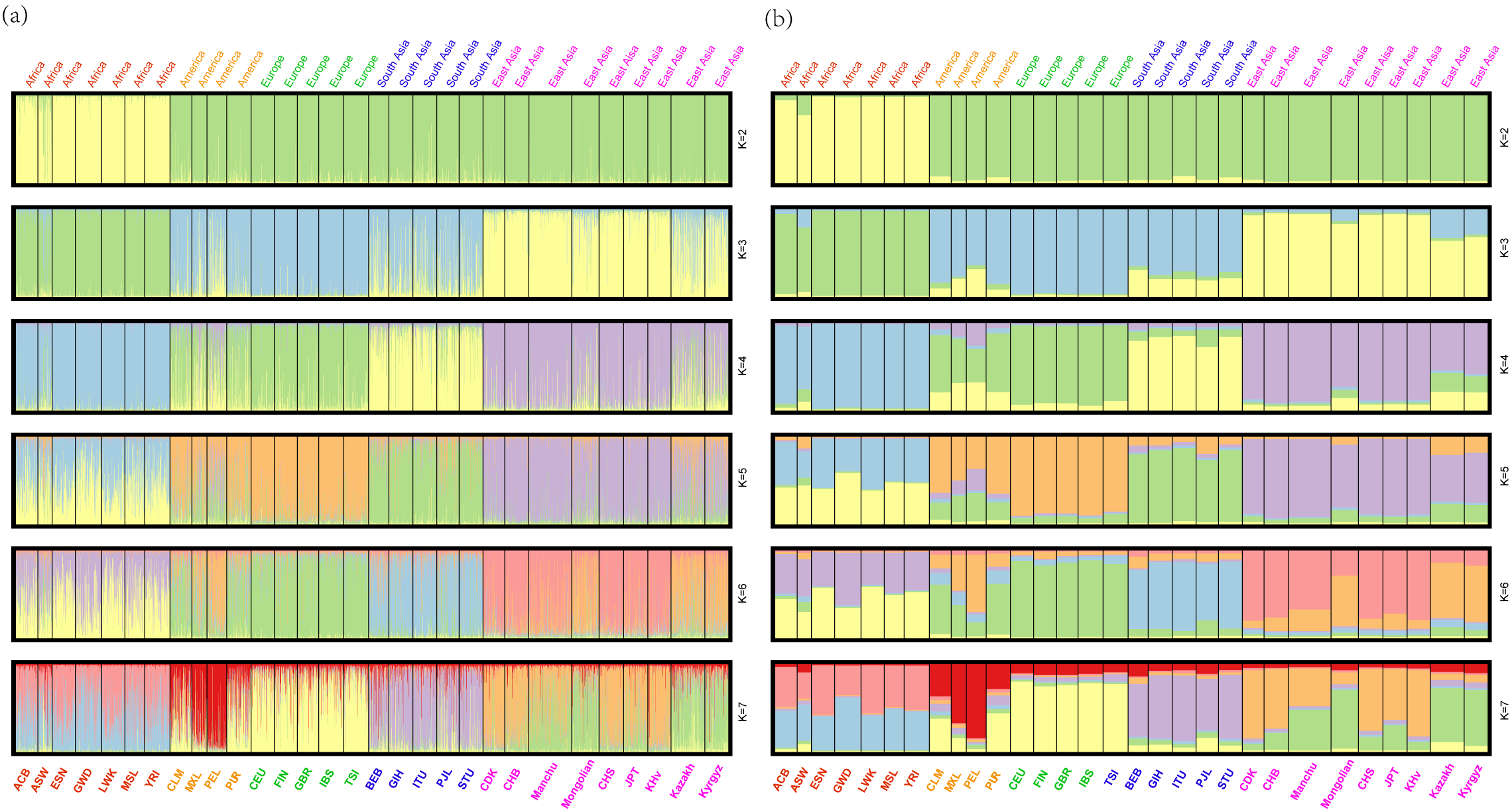

Supplement: Supplementary file 1 — Supplementary Material 1 [file 41065_2025_416_MOESM1_ESM.tif]

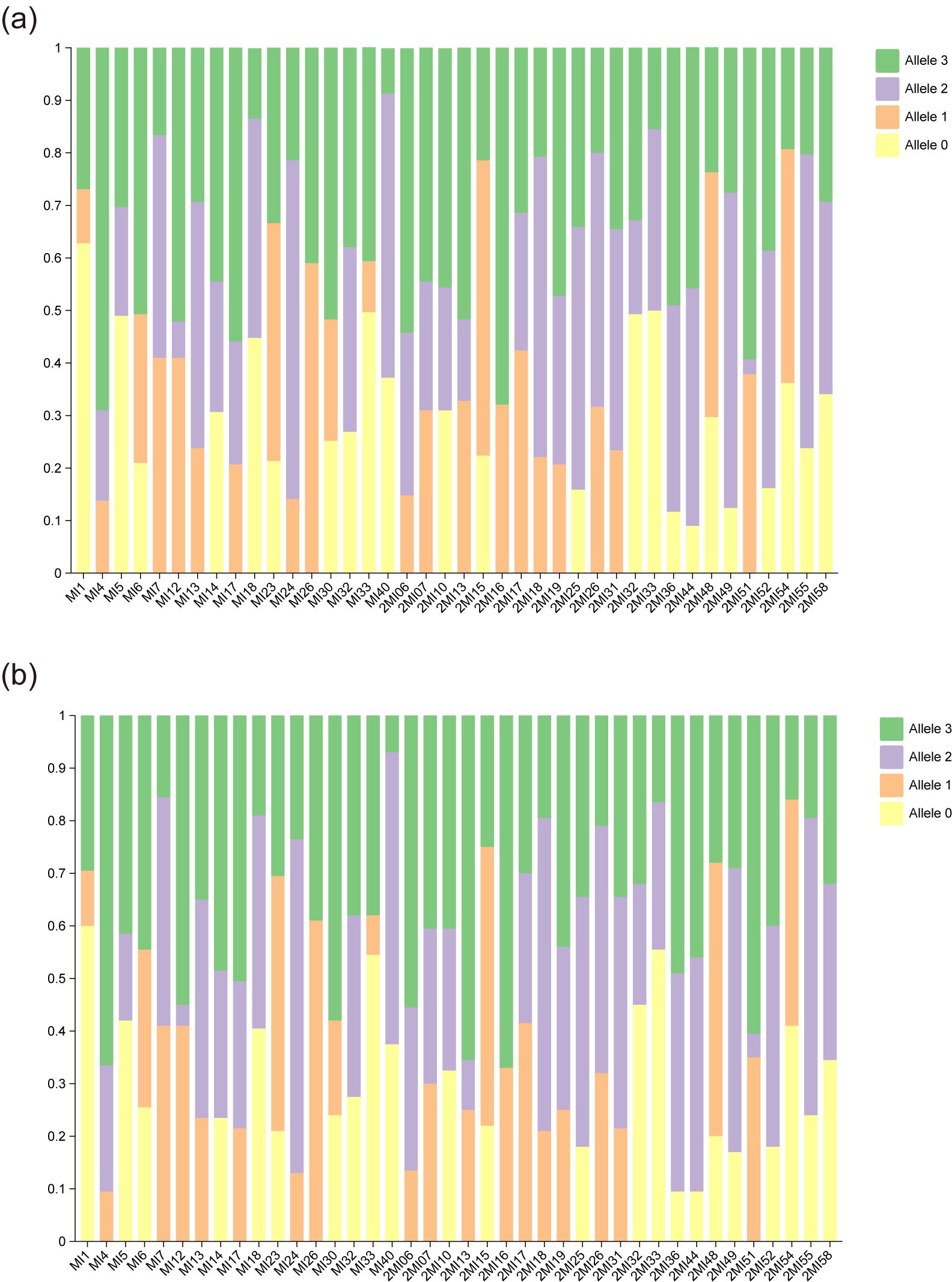

Supplement: Supplementary file 2 — Supplementary Material 2 [file 41065_2025_416_MOESM2_ESM.tif]
